# Effects of adding different levels of fructooligosaccharide to diet on productive performance, egg quality traits, immune response and blood metabolites in commercial laying hens

**DOI:** 10.1002/vms3.1550

**Published:** 2024-08-09

**Authors:** Salah Mahdi Alsherify, Ahmad Hassanabadi

**Affiliations:** ^1^ Department of Animal Production, College of Agriculture AL‐Qasim Green University Babylon Iraq; ^2^ Department of Animal Science, Faculty of Agriculture Ferdowsi University of Mashhad Mashhad Iran

**Keywords:** blood substitutes, immunology, human–animal bond

## Abstract

**Background:**

A prebiotic is defined as an indigestible feed substance that beneficially affects the host by selectively stimulating the growth and/or activity of one or a limited number of bacteria in the large intestine, thereby improving host health and products.

**Objectives:**

This study was conducted to determine the effects of supplementing prebiotic fructooligosaccharide (FOS) to the diets of Hy‐Line W‐36 laying hens.

**Methods:**

A total of 168 Hy‐Line W‐36 laying hens were allocated to four dietary levels of FOS (0, 1.0, 2.0, 3.0 g/kg diet), 6 replicates of 7 birds each during 63–74. The experiment was performed using a completely randomized design.

**Results:**

Productive performance was not significantly affected by the FOS supplementation. Body weight gain was linearly decreased with increasing FOS levels in the diet (*p* < 0.01). However, eggshell strength, shell thickness and Ca and p percentages were not significantly affected, as were anti‐sheep red blood cell titres, blood parameters and blood metabolites. In the first period of the experiment (63–65 weeks), shape index and Haugh unit at the dose of 3.0 g/kg FOS were significantly increased and decreased in comparison with control, respectively (*p* < 0.05). In the third and fourth periods (69–71 and 72–74 weeks of age), the FOS had no significant effect on the internal egg quality traits. Furthermore, FOS had a linear decrease in the most saturated fatty acids (SFAs), including myristic, palmitic, margaric and stearic fatty acids; some of the mono‐un‐SFA (MUFA; palmitoleic and ginkgolic acids), and poly‐unsaturated fatty acids (γ‐linolenic and eicosatrienoic).

**Conclusions:**

Supplementing different levels of FOS to the diet of commercial laying hens had no significant effect on the layers’ performance, immune response and blood parameters, whereas there was a significant effect on some of the internal egg quality traits and egg yolk fatty acid contents.

## Graphical abstract

Dietary fructooligosaccharide supplementation had no significant effect on the productive performance of laying hens, reduced yolk palmitoleic acid compared with control and had a linear and quadratic decrease on myristic, palmitic, stearic, palmitoleic and ginkgolic acids in egg yolk.

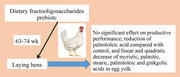

## 1 INTRODUCTION

The misuse of antibiotics in animal feed has been linked to the development of antibiotic‐resistant bacteria, posing a threat to both animal and human health. This has led to a global effort to find alternative feed additives that can promote animal health and growth without contributing to antibiotic resistance (Yang et al., 2024). According to the report by  www.marketsandmarkets.com (accessed on 20 March 2024), the feed additives market has globally increased from USD 33.0 billion in 2018 to USD 44.3 billion by 2023. Antibiotic growth promoters pose a threat to humans through animal products like meat or eggs and animals’ health as well as the environment. As a result, the European Union banned the use of antibiotics in 2006, which was followed by other countries worldwide (Yakhkeshi et al., 2011). Because of that, researchers and producers decided to use non‐antibiotic feed additives to improve production and also to prevent the adverse effects on human and animal health. Therefore, alternatives, such as probiotics, prebiotics, synbiotics, medicinal plants and organic acids, have attracted more attention during recent years. Consequently, these feed additives can enhance the host mucosa immunity and improve the resistance of pathogenic bacteria as well (Choct, 2009; Williams et al., 2001).

A prebiotic is defined as a non‐digestible food ingredient that positively affects the host growth by activating the metabolism of one or a limited number of endogenous bacteria, such as Lactobacilli and Bifidobacteria, in the intestinal tract (Gibson & Roberfroid, 1995). Prebiotic is also defined as short‐chain carbohydrates which are used as food for microorganisms in the intestinal tract and cannot be digested by pancreatic enzyme and gastric acid (Tang et al., 2015). The most dominant prebiotics that have been investigated in broilers are inulin, fructooligosaccharide (FOS), gluco‐oligosaccharides, malto‐oligosaccharides (MOS), oligofructose, stachyose and oligochitosan (Huang et al., 2007; Jiang et al., 2006; Patterson & Burkholder, 2003). Additionally, researchers may choose FOS over other feed additives due to its ability to influence the intestinal micro‐ecosystem, improve healthy microbial competition, resist pathogen adhesion and enhance metabolism and economic efficiency (Bai et al., 2024). Therefore, this study aimed to investigate the effect of different dietary doses of FOS on productive performance, immunity, blood metabolites, eggshell strength and eggshell Ca and P, internal egg quality traits and egg yolk fatty acid contents in laying hens during 63–74 weeks of age.

## 2 MATERIALS AND METHODS

### 2.1 Experimental diets and bird management

All procedures were approved by the Animal Care and Use Committee at the Ferdowsi University of Mashhad, Mashhad, Iran. A total of 168 Hy‐Line W‐36 laying hens with an initial average BW of 1.6 kg at 63 weeks of age were used in this study. The experiment was performed using a completely randomized design with four dietary levels of FOS (0, 1.0, 2.0 and 3.0 g/kg of diet) during 63–74 weeks of age for 12 weeks. The test FOS preparation (Green Spring, 95% purity) was a commercially available product that was mixed with the diet ingredients in a horizontal mixer. The birds were randomly allocated in four treatments with six replicates (experimental unit) of seven birds in each cage (replicate). Cage dimensions were 60 cm length × 60 cm width × 40 cm height. The cages were provided with two nipple drinkers and one trough feeder. During the experiment, birds received feed and water as ad libitum. The house temperature, lighting programme and relative humidity were set at 23–25°C, 16 h light:8 h dark and 60% during the experiment, respectively. Wood partitions were used to prevent cross‐feeding between the replicate cages.

Experimental diets were formulated based on the Hy‐Line W‐36 management guide for commercial layers (Hy‐Line International, 2020; Table 1). The chemical composition of the diet ingredients and the complete diet were determined based on the Association of Official Analytical Chemist (AOAC) (2019). The samples were ground and analysed for crude protein (Kjeldahl; N × 6.25; method 990.03), dry matter (method 930.15) and total ash (method 942.05). The contents of calcium (Ca) and total phosphorus (P) in the diets and eggshell samples were measured using Inductively Coupled Plasma Optical Emission Spectroscopy (ICP‐OES) instrument (SPECTRO ARCOS; method 968.08).

**TABLE 1:** Ingredients and nutrient composition of basal diets during 63–74 weeks; as‐fed basis.

Ingredients	(%)
Corn	40.86
Soybean meal (44% CP)	22.44
Wheat	20.20
Limestone	10.54
Soybean oil	3.28
Dicalcium phosphate	1.58
Common salt	0.26
NaHCO_3_	0.1
Mineral premix^a^	0.25
Vitamin premix^b^	0.25
dl‐Methionine	0.24
l‐Lysine HCL	–
Calculated values, %	
ME (kcal/kg)	2823
CP	15.24
Calcium	4.32
Available P	0.41
Digestible lysine	0.67
Digestible methionine	0.45
Digestible methionine + cystine	0.67
Sodium	0.17
Chlorine	0.20
Potassium	0.67
Dietary cation–anion balance (meq/kg)	216.3
Determined values, %	
DM	93.26
CP	15.99
Ash	13.83
Calcium	3.36
Total P	0.45

Abbreviation: DM, dry matter.

^a^ Provided per kg of diet: vitamin A (retinol), 8800 IU; vitamin D3 (cholecalciferol), 3300 IU; vitamin E (dl‐α‐tocopheryl acetate), 18.5 IU; vitamin K3 (menadione), 2.2 mg; vitamin B1 (thiamin), 2.2 mg; vitamin B2 (riboflavin), 5.5 mg; vitamin B3 (niacin), 28.0 mg; vitamin B5 (pantothenic acid), 6.6 mg; vitamin B6 (pyridoxine), 3.5 mg; vitamin B9 (folic acid), 0.7 mg; vitamin B12 (cyanocobalamin), 0.02 mg; biotin, 0.05 mg; antioxidant 1.0 mg.

^b^ Provided (mg/kg of diet): Mn (manganese sulphate) 80.0, Fe (iron sulphate) 75.0, Zn (zinc sulphate) 64.0, Cu (copper sulphate) 6.0, Se (sodium selenite) 0.3.

### 2.2 Sample collection

Eggs were collected daily at 8:00 AM. Then, they were weighed using a digital scale (0.001 g digital scale, model GF 400, A&D Weighing). Egg production and the egg mass were calculated daily based on the following formulas:

Egg production (%) = number of eggs per replicate/number of birds per replicate × 100
Egg mass (g/hen/day) = egg weight per replicate × egg production % per replicate/100

Daily FI was measured every 3 weeks by taking the difference of given feed at the beginning and remaining feed in the feeders at the end of the period and then divided by 21. The FCR was calculated by dividing FI (g) over the egg mass (g; Bakhshalinejad et al., 2018). No mortality occurred during the experiment.

### 2.3 Blood parameter measurements

On the last day of the study, one bird from each replicate was randomly selected. About 5 mL of blood was taken from the wing vein and placed into a vacuum tube. Blood samples were kept at room temperature for about 2 h until clotting and centrifuged at (3000 *g*; 10 min; 4°C). The collected serum was stored at −20°C for further analysis. Biochemical analyses were performed according to the standard protocol using commercial laboratory kits (Parsazmoon). Glass tubes containing ethylenediaminetetraacetic acid (EDTA) were used for blood collecting to determine the levels of haematocrit (HCT), haemoglobin (HGB), red blood cells (RBC), white blood cells (WBCs), phosphorus (P), calcium (Ca), triglycerides (TG), total cholesterol (Chol), low‐density lipoprotein cholesterol (LDL‐C) and high‐density lipoprotein cholesterol (HDL‐C). Blood samples were analysed using Hematoanalyzer (model KX21, Sysmex Corporation). All samples were analysed in duplicate and done immediately to avoid variations.

### 2.4 Humoral immune response

To assay the humoral immune response against sheep RBCs (SRBCs), 10 mL of blood sample was taken from a ram and shed in a glass tube containing EDTA. The red cells were washed three times with phosphate‐buffered saline (PBS) solution and then a 5% solution of RBC was prepared in saline phosphate buffer. For clarifications, all the steps above were done in sterile conditions. At 71 weeks of age, one hen per replicate was injected with 0.5 mL of SRBC in the wing vein. A colour spray was used to differentiate between injected and non‐injected birds (Alsherify et al., 2022). To measure the primary and secondary antibody responses against SRBC, 2 mL of blood sample was taken from the wing vein 7 days after the first injection. After blood clotting, serum was removed by centrifuge (3000 × *g*; 10 min; 4°C). Collected sera were placed for 0.5 h at 56°C for measurement of total anti‐SRBC titres, IgG and IgM. The titres of antibody were presented as the log2 of the highest dilution level of serum that agglutinated 0.05 mL of 2.5% suspension of SRBC in PBS (Eftekhari et al., 2018).

### 2.5 Eggshell strength

At the end of the experiment, one egg was randomly selected from each replicate (six eggs per treatment). The eggs were transferred to the laboratory. Eggshell strength was determined using a strength metre (model H5KS, Tinius Olsen Co.) with a maximum power of 50 N and a speed of 10 mm per minute (Bakhshalinejad et al., 2018). The egg was placed on its side (horizontally), and the maximum force imposed by the impact of a falling steel rod on eggshells was recorded (Bakhshalinejad et al., 2018).

### 2.6 Eggshell calcium and phosphorus

To determine the concentration of calcium and total phosphorus in the eggshells, 1 egg was randomly selected from each replicate (6 eggs per treatment, 24 eggs in total,) on the last day of the study. The eggs were kept at 4°C for further analysis. On the day of analysis, the eggs were individually broken, and the shell was separated from the yolk and albumen. Eggshells were then washed with distilled water and left 48 h to dry at room temperature. After drying, the eggshells were ground and placed in a separate plastic bag specified for each replicate. Finally, Ca and P concentrations in the eggshells were measured using a spectrophotometer (ICP‐OES, SPECTRO ARCOS Co.) with standard methods (AOAC, 2019).

### 2.7 Body weight changes

The hens were weighed by a digital scale at the beginning and at the end of the experiment by randomly taking three replicates from each treatment (Bakhshalinejad et al., 2018).

### 2.8 Statistical analyses

All data were tested for normality by SAS 9.4 software through the univariate plan normal procedure (Statistical Analysis System [SAS], 2012) prior to the statistical analysis. Then, the data were analysed using PROC GLM of SAS software (SAS, 2012) for variance analyses in a completely randomized design. Duncan's multiple range test was applied to separate treatment means (*p* < 0.05). PROC REG was used to test linear and quadratic responses to increasing dietary levels of FOS.

## 3 RESULTS

### 3.1 Productive performance

The productive performance, including the percentage of egg production, egg mass and FI, was not significantly affected by the dietary levels of FOS powder during this trial; except that FCR during 69–71 weeks was significantly affected in the third period, FCR also increased linearly in this period (*p* ≤ 0.05) with increasing FOS levels in the diet (Table 2). Polynomial tests showed that none of the productive performance parameters of laying showed a linear or quadratic response to the treatments in any of the experimental periods (Table 2; *p* > 0.05).

**TABLE 2:** Effect of different dietary supplementation levels of prebiotic (fructooligosaccharide) on productive performance parameters of Hy‐Line W‐36 laying hens during 63–74 weeks of age.

Prebiotic level	Period 1	Period 2	Period 3	Period 4	Total
(g/kg diet)	63–65 weeks	66–68 weeks	69–71 weeks	72–74 weeks	63–74 weeks
	**Egg production (%)**				
0 (control)	84.92	83.56	79.59	80.16	82.06
1	87.42	85.60	81.86	79.36	83.56
2	87.87	85.72	82.09	84.01	84.92
3	85.71	85.37	79.82	79.02	82.48
SEM	0.34	0.35	0.35	0.40	0.32
	*p*‐Value				
ANOVA	0.577	0.819	0.669	0.452	0.530
Linear	0.160	0.410	0.221	0.405	0.169
Quadratic	0.175	0.510	0.214	0.403	0.187
	**Egg mass (g/hen/day)**				
0 (control)	55.43	54.55	52.22	52.47	53.67
1	57.29	56.19	53.68	52.31	54.87
2	56.85	55.93	53.60	55.10	55.37
3	56.09	55.93	52.15	51.85	54.00
SEM	0.29	0.30	0.29	0.34	0.27
	*p*‐Value				
ANOVA	0.731	0.800	0.708	0.522	0.666
Linear	0.281	0.420	0.264	0.372	0.219
Quadratic	0.297	0.521	0.239	0.370	0.234
	**Feed intake (g/hen/day)**				
0 (control)	103.0	102.6	97.4	98.9	100.5
1	107.2	103.3	93.4	97.3	100.3
2	105.9	108.8	97.8	105.2	104.4
3	102.8	108.5	102.3	101.3	103.7
SEM	0.39	0.52	0.48	0.49	0.41
	*p*‐Value				
ANOVA	0.451	0.549	0.368	0.444	0.553
Linear	0.146	0.610	0.416	0.573	0.654
Quadratic	0.116	0.893	0.227	0.751	0.916
	**Feed conversion ratio (g:g)**				
0 (control)	1.86	1.88	1.87^ab^	1.89	1.88
1	1.88	1.85	1.74^b^	1.87	1.83
2	1.86	1.95	1.82^ab^	1.92	1.89
3	1.84	1.94	1.96^a^	1.96	1.92
SEM	0.05	0.07	0.06	0.07	0.06
	*p*‐Value				
ANOVA	0.863	0.707	0.050	0.852	0.608
Linear	0.688	0.975	0.050	0.855	0.593
Quadratic	0.548	0.823	0.019	0.676	0.407

^a,b^Means without common superscript within a column are significantly different (*p* < 0.05).

### 3.2 Blood metabolites and immune response

FOS supplementation did not have any significant effect on blood parameters of RBC, WBC, HGB and HCT. Moreover, no significant effects were observed on blood biochemical characteristics of TG, Chol, HDL‐C, LDL‐C, HDL‐C‐to‐LDL‐C ratio, Ca and P during this study. In addition, total anti‐SRBC titre, IgG and IgM were not significantly affected by the dietary levels of FOS powder (Table 3). Polynomial tests did not reveal significant linear or quadratic responses of the blood serum metabolites in response to dietary FOS supplementation levels at any of the experimental periods (Table 3).

**TABLE 3:** Effect of different dietary supplementation levels of prebiotic (fructooligosaccharide) on blood serum metabolites and antibody titres in Hy‐Line W‐36 laying hens at the end of 74 weeks of age.

Prebiotic level (g/kg diet)	TG (mg/dL)	Chol (mg/dL)	HDL‐C (mg/dL)	LDL‐C (mg/dL)	HDL‐C:LDL‐C ratio	Ca (mg/dL)	P (mg/dL)
0 (control)	1293.8	199.7	67.50	59.33	1.18	20.37	6.25
1	1324.2	172.5	76.50	59.83	1.36	21.33	6.00
2	1395.3	205.5	68.83	58.83	1.39	20.55	6.40
3	1246.5	178.7	62.67	61.67	1.04	20.38	5.90
SEM	3.53	1.00	0.63	0.65	0.12	0.23	0.14
	*p*‐Value						
ANOVA	0.949	0.345	0.434	0.989	0.644	0.799	0.558
Linear	0.654	0.895	0.324	0.906	0.291	0.530	0.779
Quadratic	0.621	0.992	0.205	0.851	0.226	0.474	0.655
	RBC **(10^12^/L)**	**WBC (10^9^/L)**	**HGB (g/dL)**	**HCT** **(%)**			
0 (control)	2.44	10.76	11.95	32.33			
1	2.39	10.95	11.73	31.67			
2	2.40	10.78	12.40	32.00			
3	2.38	10.68	11.87	31.33			
SEM	0.08	0.15	0.15	0.29			
	*p*‐Value						
ANOVA	0.968	0.944	0.541	0.943			
Linear	0.830	0.726	0.609	0.887			
Quadratic	0.925	0.652	0.650	1.00			
	**Total Ig**	**IgG**	**IgM**				
		(log2)					
0 (control)	8.83	8.17	0.67				
1	7.67	7.00	0.67				
2	8.83	8.33	0.50				
3	8.00	6.67	1.33				
SEM	0.20	0.20	0.15				
	*p*‐Value						
ANOVA	0.421	0.161	0.265				
Linear	0.697	0.955	0.369				
Quadratic	0.791	0.703	0.190				

Abbreviations: Ca, calcium; Chol, total cholesterol; HCT, haematocrit; HDL‐C, high‐density lipoprotein cholesterol; HGB, haemoglobin; LDL‐C, low‐density lipoprotein cholesterol; P, phosphorus; RBC, red blood cells; TG, triglycerides; WBCs, white blood cells.

### 3.3 Eggshell characteristics and body weight changes

Eggshell strength and the eggshell Ca and P percentages were not significantly affected by the studied factors. However, body weight gain (BWG) was significantly affected by the FOS levels, and it was also linearly decreased (*p* < 0.05) by increasing FOS levels in the diet of laying hens (Table 4). At the same time, except for BWG, which showed a linear decrease, none of the other aforementioned traits showed a significant linear or quadratic response to dietary FOS supplementation levels at 74 weeks of age (Table 4).

**TABLE 4:** Effect of different dietary supplementation levels of prebiotic (fructooligosaccharide) on body weight gain (BWG), eggshell strength and shell Ca and P percentages in Hy‐Line W‐36 laying hens at the end of 74 weeks of age.

Prebiotic level (g/kg)	BWG (g/hen)	Eggshell strength (N)	Ca (%)	P (%)
0 (control)	100.0^a^	36.25	38.06	0.16
1	85.11^b^	33.25	36.14	0.08
2	47.62^c^	37.97	37.72	0.14
3	85.71^b^	34.75	38.32	0.15
SEM	4.0	0.40	0.25	0.05
	*p*‐Value			
ANOVA	0.000	0.538	0.370	0.359
Linear	0.036	0.960	0.270	0.228
Quadratic	0.085	0.963	0.192	0.201

^a,c^ Means without common superscript within a column are significantly different (p < .05).

### 3.4 Egg quality traits

Egg quality parameters were measured four times during the experimental period of 63–74 weeks, and each period lasted for 3 weeks. Table 5 shows the effect of FOS on egg quality traits in Hy‐line W‐36 laying hens during 63–74 weeks of age. FOS did not show any significant effect on the internal egg quality measurements, including egg weight, egg specific gravity, shell thickness, yolk colour index and the percentage of yolk, albumin and shell, except for the shape index and Haugh unit. The shape index was significantly (*p* < 0.05) increased by increasing FOS levels in the diet. But, the Haugh unit was significantly decreased by increasing (*p* < 0.05) the FOS concentration in the diet of laying hens.

**TABLE 5:** Effect of different dietary supplementation levels of prebiotic (fructooligosaccharide) on the internal egg quality traits in Hy‐Line W‐36 laying hens in different ages.

Prebiotic level (g/kg diet)	Egg weight (g)	Egg specific gravity	Shape index (%)	Haugh unit	Yolk colour index	Shell thickness (mm)	Yolk (%)	Albumin (%)	Shell (%)
	**63–65 weeks**								
0 (control)	68.05	1.09	76.59^b^	100.38^a^	103.7	0.30	25.55	65.52	8.93
1	66.41	1.09	77.99^a^	94.50^ab^	103.8	0.29	25.26	65.82	8.92
2	66.36	1.08	75.92^b^	94.63^ab^	103.7	0.29	26.36	64.60	9.04
3	66.25	1.08	78.27^a^	88.40^b^	103.5	0.31	26.81	63.88	9.30
SEM	0.29	0.01	0.16	0.42	0.12	0.03	0.22	0.21	0.12
	*p*‐Value								
ANOVA	0.684	0.585	0.001	0.028	0.724	0.667	0.419	0.164	0.572
Linear	0.370	0.441	0.630	0.415	0.564	0.356	0.949	0.880	0.808
Quadratic	0.524	0.621	0.391	0.944	0.414	0.260	0.605	0.431	0.523
	**66–68 week**								
1	63.95	1.085	77.28	89.16	103.33	0.29	26.22	64.54	9.24
2	66.32	1.084	78.04	91.13	103.50	0.31	25.72	64.97	9.30
3	65.28	1.082	77.62	93.67	103.50	0.28	25.60	65.03	9.37
4	65.33	1.084	77.89	92.82	103.33	0.30	26.35	64.39	9.27
SEM	0.28	0.01	0.21	0.44	0.12	0.26	0.21	0.22	0.15
	*p*‐Value								
ANOVA	0.537	0.526	0.844	0.680	0.898	0.069	0.831	0.892	0.991
Linear	0.255	0.215	0.609	0.426	0.460	0.819	0.391	0.483	0.767
Quadratic	0.313	0.256	0.698	0.611	0.441	0.816	0.358	0.441	0.782
	**69–71 weeks**								
0 (control)	65.32	1.085	76.65	90.64	103.50	0.30	26.02	64.15	9.82
1	65.74	1.084	77.71	89.56	103.50	0.28	26.74	64.50	8.76
2	65.53	1.084	77.64	89.03	103.50	0.28	25.37	65.65	8.98
3	65.77	1.087	77.71	86.50	103.67	0.30	25.90	64.30	9.79
SEM	0.25	0.01	0.20	0.46	0.12	0.29	0.25	0.26	0.15
	*p*‐Value								
ANOVA	0.984	0.280	0.545	0.815	0.933	0.367	0.757	0.688	0.087
Linear	0.860	0.230	0.273	0.967	0.826	0.082	0.977	0.349	0.016
Quadratic	0.920	0.113	0.418	0.814	0.703	0.079	0.923	0.385	0.012
	**72–74 weeks**								
0 (control)	67.90	1.081	77.46	89.96	104.50	0.29	26.49	64.61	8.90
1	68.36	1.080	77.57	87.79	104.17	0.27	25.56	65.86	8.58
2	68.91	1.082	78.02	92.15	104.17	0.29	25.39	66.10	8.52
3	69.59	1.079	78.04	91.67	104.00	0.27	25.48	65.60	8.92
SEM	0.31	0.01	0.19	0.41	0.12	0.29	0.22	0.23	0.15
	*p*‐Value								
ANOVA	0.843	0.691	0.807	0.613	0.404	0.454	0.657	0.564	0.732
Linear	0.856	0.755	0.723	0.938	0.390	0.705	0.316	0.186	0.278
Quadratic	0.934	0.631	0.927	0.742	0.687	0.816	0.457	0.264	0.260

^a,b^ Means without common superscript within a column are significantly different (p < .05).

In the second (66–68 weeks), third (69–71 weeks) and fourth (72–74 weeks) experimental periods, no significant effect of FOS was found on the internal and external egg quality traits except that shell percentage showed a significant linear and quadratic trend in response to dietary FOS levels in third period (Table 5).

### 3.5 Fatty acid profile

Saturated fatty acids (SFA) of myristic, palmitic, margaric and stearic showed significant linear and quadratic trends (*p* < 0.05) in response to the FOS levels in the diet, except for the tricosylic fatty acid (C23:0), which was not significantly affected by the FOS levels (Table 6).

**TABLE 6:** Effect of different dietary supplementation levels of prebiotic (fructooligosaccharide) on saturated fatty acid content of egg yolk (mg FA/g egg yolk) in Hy‐Line W‐36 laying hens at the end of 74 weeks of age.

Prebiotic level	C14:0	C16:0	C17:0	C18:0	C23:0	SFA
(g/kg)	Myristic	Palmitic	Margaric	Stearic	Tricosylic	
0 (control)	4.90	255.52	2.00	98.79	9.07	146.94
1	3.01	236.00	1.87	80.75	9.04	137.62
2	2.67	239.44	1.57	78.21	8.72	145.27
3	3.36	254.21	1.84	80.99	9.25	157.22
SEM	0.20	1.46	0.12	0.73	0.23	5.39
	*p*‐Value					
ANOVA	0.058	0.958	0.552	0.250	0.972	0.773
Linear	<0.0001	0.001	0.016	<0.0001	0.306	0.417
Quadratic	<0.0001	0.003	0.046	0.002	0.485	0.457

Abbreviation: SFAs, saturated fatty acids.

As shown in Table 7, palmitoleic fatty acid was the only monoun SFA that was significantly affected by the increasing FOS levels. Other mono‐unsaturated fatty acids (MUFAs), including palmitoleic, ginkgolic and oleic fatty acids, showed a significant linear response, whereas palmitoleic and ginkgolic showed quadratic (*p* < 0.05) response; however, other fatty acids (C18:1n9t and C20:1) were not significantly affected by the FOS levels. Moreover, the oleic fatty acid (C18:1n9c) showed a linear significant (*p* < 0.05) effect due to increasing FOS levels, so that the amount of this fatty acid was linearly decreased by increasing the FOS levels in the diet. The optimum level of FOS was the control or 3 g FOS per kg diet, due to their highest MUFA values compared to FOS in doses of 1 and 2 g/kg FOS in the diet.

**TABLE 7:** Effect of different dietary supplementation levels of prebiotic (fructooligosaccharide) on mono‐unsaturated fatty acid (MUFA) content of egg yolk (mg FA/g egg yolk) in Hy‐Line W‐36 laying hens at the end of 74 weeks of age.

Prebiotic level	C16:1	C17:1	C18:1n9t	C18:1n9c	C20:1	MUFA
(g/kg)	Palmitoleic	Ginkgolic	Elaidic	Oleic	Eicosenoic	
0 (control)	29.05^a^	0.93	1.46	416.26	2.18	447.55
1	20.44^b^	0.68	1.39	380.68	2.19	402.96
2	22.13^b^	0.70	1.52	369.60	2.09	393.83
3	24.17^ab^	0.90	1.53	380.57	1.97	407.18
SEM	0.38	0.08	0.12	1.43	0.11	13.0
	*p*‐Value					
ANOVA	0.050	0.231	0.968	0.712	0.829	0.651
Linear	<0.0001	0.006	0.377	0.034	0.610	0.260
Quadratic	0.000	0.012	0.502	0.135	0.973	0.363

^a,b^ Means without common superscript within a column are significantly different (p < .05).

All the polyunsaturated fatty acids (PUFA) were not significantly affected by the FOS levels except for γ‐linolenic and eicosatrienoic fatty acids that showed a linear and quadratic trends (*p* < 0.05) in response to FOS levels in diet (Table 8). The optimum level was 3 g FOS/kg diet due to its highest values compared to other treatments.

**TABLE 8:** Effect of different dietary supplementation levels of prebiotic (fructooligosaccharide) on polyunsaturated fatty acid (PUFA) content of egg yolk (mg FA/g egg yolk) in Hy‐Line W‐36 laying hens at the end of 74 weeks of age.

Prebiotic level (g/kg diet)	C18:2n6c	C18:3n3	C18:3n6	C20:2	C20:3n6	C20:4n6	C22:6n3				
	Linoleic	α‐Linolenic	γ‐Linolenic	Eicosadienoic	Eicosatrienoic	Arachidonic	Docosahexaenoic	PUFA	n3	n6	n6/n3
0 (control)	135.09	2.59	0.94	1.35	2.00	9.07	4.54	152.07	7.13	146.94	21.04
1	126.5	2.34	0.67	1.16	1.54	9.04	4.93	143.3	7.27	137.6	19.29
2	134.0	2.38	0.81	1.23	1.71	8.72	4.76	150.7	7.13	145.3	21.43
3	145.2	2.82	0.94	1.54	1.85	9.25	5.09	163.3	7.91	157.2	20.30
SEM	0.91	0.14	0.09	0.10	0.11	0.23	0.20	5.67	0.35	5.39	0.49
					*p*‐Value						
ANOVA	0.761	0.660	0.351	0.383	0.363	0.972	0.928	0.789	0.907	0.773	0.614
Linear	0.165	0.075	0.008	0.154	0.004	0.306	0.808	0.581	0.849	0.555	0.808
Quadratic	0.193	0.093	0.007	0.131	0.008	0.485	0.919	0.441	0.712	0.419	0.802

## 4 DISCUSSION

### 4.1 Productive performance

Productive performance, including egg production, egg mass, feed intake and FCR, were not significantly affected by the increasing levels of FOS in the current study, which agreed with the results reported by Mohebbifar et al. (2013), who concluded that all the productive performance was not significantly affected by supplementing prebiotics to the diets. In addition, Świątkiewicz et al. (2010) found that using oligofructoses as a prebiotic feed additive had no significant effect on the egg production percentage, egg mass, feed intake and FCR. Moreover, Jahanian Najafabadi et al. (2017) reported that laying hens’ performance, including egg weight, egg production, egg mass, feed intake and FCR, were not significantly affected by MOS prebiotics. However, other studies (Abdelqader et al., 2013; Jahanian & Ashnagar, 2015; Li et al., 2007; Xu et al., 2020; Zhou et al., 2021) found that both egg production and egg mass were positively affected by prebiotics such as FOS.

FOSs and monosaccharides share some similarities in their characteristics and functionality. Both are carbohydrates, with FOS being a type of oligosaccharide (Kherade, 2021). They are both used in the food industry, with FOS being incorporated into various products due to its functional properties and health benefits (Kherade, 2021; Xiang‐ting, 2006). In terms of analysis, both FOS and monosaccharides can be separated and analysed using gas liquid chromatography and high‐performance liquid chromatography (Hagiwara et al., 1983). In addition, in the current study, FOS decreased FI and increased FCR, whereas oligofructosaccharides improved FCR with no effect on the FI in laying hens, as reported by Li et al. (2007) and Chen et al. (2005), which disagreed with the results obtained in this study. Moreover, Chen et al. (2005) reported that the inclusion of oligofructose in the laying hens’ diet positively affects egg production and FCR.

The efficacy of FOS on laying hens’ performance is attributed to its ability to improve egg production, feed conversion ratio and egg quality traits, as demonstrated in several studies (Kim et al., 2009; Sjofjan et al., 2020; Yassein et al., 2015). These improvements are likely due to the prebiotic properties of FOS, which promote the growth of beneficial gut bacteria, leading to better nutrient absorption and overall health. Additionally, FOS has a positive impact on serum immunoglobulin levels and small intestinal microflora in laying hens (Kim et al., 2009).

### 4.2 Blood parameters

Blood parameters, including RBC, WBC, HGB and HCT, were not significantly affected by the FOS levels in the diet, which agreed with the results reported by Mohebbifar et al. (2013), who observed that blood parameters were not significantly affected by the prebiotic treatments. In addition, Zarei et al. (2011) found that WBC count was not affected by prebiotic levels, which is similar to our results.

No significant effects were found for FOS levels on blood biochemical traits. These results agreed with the results reported by Zarei et al. (2011) when they reported that prebiotic had no significant effect on TG, Chol and HDL‐C in laying hens. These results disagreed with the results reported by Kalani et al. (2022), when they found that dietary prebiotics decreased TG, Chol and LDL‐C in the blood of the laying hens.

### 4.3 Egg quality traits and immune response

Egg quality traits, in this study, were not affected by prebiotic levels of FOS. These results are similar to those reported by Mohebbifar et al. (2013), who found that the quality traits of eggs, including egg weight, yolk colour, Haugh unit, shell weight and shell thickness, were not significantly affected by supplementing prebiotics to the laying hens’ diets. In addition, some authors (Jahanian & Ashnagar, 2015; Li et al., 2007; Shang et al., 2010) observed that FOS, MOS and inulin prebiotics had no significant effect on eggshell thickness, which is similar to our results. Similarly, Jahanian Najafabadi et al. (2017) found that egg quality traits, including Haugh unit, shell weight, shell thickness, specific gravity and yolk colour, were not significantly affected by prebiotic feed additives. Moreover, Świątkiewicz et al. (2010) found that the dietary application of oligofructose as a prebiotic had no significant effect on the egg weight and eggshell thickness. In addition, Tang et al. (2015) reported that prebiotics had no remarkable effect on Haugh units, albumin and yolk contents, shell thickness and egg‐specific gravity. Moreover, yolk colour was not significantly effected by FOS prebiotic (Li et al., 2007). Several studies also did not show any significant effect of prebiotics on the eggshell thickness and egg specific gravity (Kurtoglu et al., 2004; Li et al., 2007; Yi et al., 2014). However, Obianwuna et al. (2022) found a remarkable effect of FOS on the eggshell thickness, yolk colour and Haugh unit, which is not similar to the results of this study.

The immune system was not affected by FOS levels in the diet. However, Obianwuna et al. (2022) found that FOS enhanced the immune system in the laying hens, which is not similar to our results. In addition, Kalani et al. (2022) reported that prebiotics can reduce the colonization of pathogenic bacteria such as *Salmonella enterica* in the ceca of laying hens.

### 4.4 Eggshell characteristics and body weight

FOS had no significant effect on the eggshell strength, which agrees with the results published by Mohebbifar et al. (2013), who found that prebiotic supplementation in the laying hens’ diets had no significant effect on their body weight change. However, Xu et al. (2003) reported that using FOS at the level of 4 g/kg diet significantly improved the BWG compared with the control group in the laying hens, which is disagreed with our results. Moreover, Obianwuna et al. (2022) found a similar result when they reported that FOS had no noteworthy effect on the eggshell strength. In the current study, eggshell percentage during the period of 69–71 weeks showed a linear and quadratic trend against dietary supplementation levels of FOS. The reason for this trend is not clear to the authors. In particular, it decreased at the levels of 1 and 2 g/kg of FOS compared to the control and then increased at the level of 3 g FOS/kg diet, and its percentage reached almost the same as the control treatment.

FOS did not significantly affect BWG in hens due to various probable factors. A research indicated that, in turkeys, different levels of FOS (ranging from 0.5% to 2%) did not impact body weight when added to their diets for 8 weeks (Jankowski et al., 2005). Similarly, in broilers, FOS supplementation did not affect growth performance over a 5‐week period (Yang et al., 2022). Additionally, a study on the simultaneous use of FOS with competitive exclusion cultures in the diet showed no consistent differences in BWG compared to controls in broiler chicks challenged with salmonella (Telg & Caldwell, 2009). Muramatsu et al. (1993) also noted that fructose, a component of FOS, increased lower gut weights in chickens, indicating a potential physiological effect. These findings suggest that although FOS may not directly affect weight gain in hens, it may have other beneficial effects on their health and productivity. The lack of significant impact on BWG could be attributed to the specific doses of FOS used, the duration of supplementation and the overall diet composition, highlighting the complexity of FOS effects on body weight in poultry.

### 4.5 Egg yolk fatty acids

Throughout the literature, not much research has been done on the effect of prebiotics on egg yolk fatty acids. Therefore, it is difficult to find a better explanation and compare it with our results.

SFAs of myristic, palmitic, margaric and stearic showed a significant linear and quadratic response to the FOS levels in the diet, except for the tricosylic fatty acid (C23:0), which was not significantly affected by the FOS levels. SFAs have a major effect on the human health throughout the blood pressure and coronary heart disease (Tang et al., 2015). In our study, it was found that FOS decreased most of the SFAs in comparison with the control group, which is promising even though it was not significant. Therefore, it is recommended to use higher levels of this probiotic in the diet of laying hens in future studies. Similar result was found by Tang et al. (2015) when they reported that prebiotics had no significant effect on the egg yolk fatty acid composition in young laying hens; however, prebiotic had a significant effect on the SFAs in the older hens, which is not consistent with the results of the present study.

Palmitoleic fatty acid was the only monoun SFA affected by FOS levels, whereas it was linearly increased by increasing the levels of FOS in comparison with the control. Other MUFAs, including palmitoleic and ginkgolic fatty acids (C16:1 and C17:1), showed a significant linear and quadratic response, whereas the other fatty acids (C18:1n9t and C20:1) were not significantly affected by the FOS levels. Moreover, the oleic fatty acid (C18:1n9c) showed a linear significant response to FOS levels; however, the amount of this fatty acid was linearly decreased by increasing levels of FOS in the diet.

All the PUFAs were not significantly affected by the FOS levels except for γ‐linolenic and eicosatrienoic fatty acids, which showed significant linear and quadratic responses. This result is disagreed with the results reported by Tang et al. (2015) when they reported that prebiotics can increase the unsaturated fatty acid levels, including linoleic, α‐linolenic and *n*‐6 fatty acids. In addition, FOS had increased α‐linolenic acid level in the egg yolk, as reported by Yi et al. (2014), which is contrary to the results of the present study.

In this study, the addition of FOS to the diet of laying hens did not have a significant effect on the total concentration of SFA, MUFA and PUFA in egg yolk; but some individual SFAs, such as myristic, palmitic, margaric and stearic, MUFAs of palmitoleic and ginkgolic and PUFAs of γ‐linolenic and eicosatrienoic had a linear and quadratic trend against dietary supplementation levels of FOS. The reason for this trend is not clear to the authors. In particular, they were decreased at the levels of 1 and 2 g/kg of FOS compared to the control and then increased at the level of 3 g FOS/kg diet, and their concentrations in the egg yolk was reached almost the same as in the control treatment. Therefore, the optimum level of FOS according to the concentration of palmitoleic and gingcolic fatty acids in egg yolk was 0 or 3 g/kg FOS in the diet due to their highest amounts compared to FOS in doses of 1 and 2 g/kg FOS in the diet.

It has been shown that the inclusion of FOS in the diet of laying hens has a significant effect on the fatty acid composition of eggs. Rakonjac et al. (2023) found that the highest content of SFA was in organic New Hampshire eggs, whereas the highest content of MUFA was in organic Isa Brown eggs, and the highest content of PUFA was in floor‐produced eggs. This suggests a potential relationship between FOS and the fatty acid composition of eggs. In addition, FOS plays a significant role in enhancing the fatty acid composition of the eggs. It has been found that FOS positively modulates fat deposition, lipid metabolism, reproductive hormones and adipokines in hens, leading to improved egg production and quality (Wen et al., 2022). Furthermore, FOS utilization has been linked to an increase in the relative abundance of specific beneficial bacteria like *Bifidobacterium pseudolongum* in the gut, which can further impact the fatty acid composition of eggs (Yi et al., 2014).

## 5 CONCLUSIONS

Dietary supplementation of FOS did not significantly affect laying hens’ performance (egg production, egg mass, feed intake, FCR), blood metabolites, eggshell strength, shell calcium, and phosphorus, and immunity. With the increase of FOS in the diet, the egg shape index increased and the Haugh unit decreased. Most of the saturated and monoun SFAs showed a significant linear and quadratic decrease in response to FOS supplementation levels. It can be concluded that the use of FOS in the laying hens’ diet did not have that much significance on the laying hen's performance.

## AUTHOR CONTRIBUTIONS

**Salah Mahdi Alsherify**: Investigation; data curation; software; formal analysis; writing – original draft. **Ahmad Hassanabadi**: Project administration; methodology; conceptualisation; funding acquisition; writing – review and editing.

## CONFLICT OF INTEREST STATEMENT

The authors confirmed that there were no conflicts of interest.

## ETHICS STATEMENT

All procedures were approved by the Animal Care and Use Committee of the Ferdowsi University of Mashhad, Mashhad, Iran.

### PEER REVIEW

The peer review history for this article is available at https://publons.com/publon/10.1002/vms3.1550.

## ANIMAL WELFARE STATEMENT

The authors confirm that the journal's ethical policies, as outlined on the journal's author guidelines page, have been followed and the approval of the relevant ethical review committee has been obtained. The authors confirm that they have followed EU standards for the protection of animals used for scientific purposes.

As part of this experiment, all animal procedures and ethics considerations were performed following the Guide to the Care and Use of Agricultural Animals in Research and Teaching (FASS, 2010). Moreover, this study was conducted according to the procedures established by the Iranian Ministry of Agriculture (Experimental Authorization No. ASRI‐2016‐95014).

## Data Availability

The data that support the findings of this study are available from the corresponding author upon reasonable request.
